# Supplementary material for: Comparative Analysis of Herbaceous and Woody Cell Wall Digestibility by Pathogenic Fungi
Source: Molecules. 2021 Nov 28;26(23):7220. doi: 10.3390/molecules26237220 (PMC8659149; doi:10.3390/molecules26237220)
Supplement: Supplementary file 1 [file molecules-26-07220-s001.zip › molecules-1453707-supplementary.pdf]

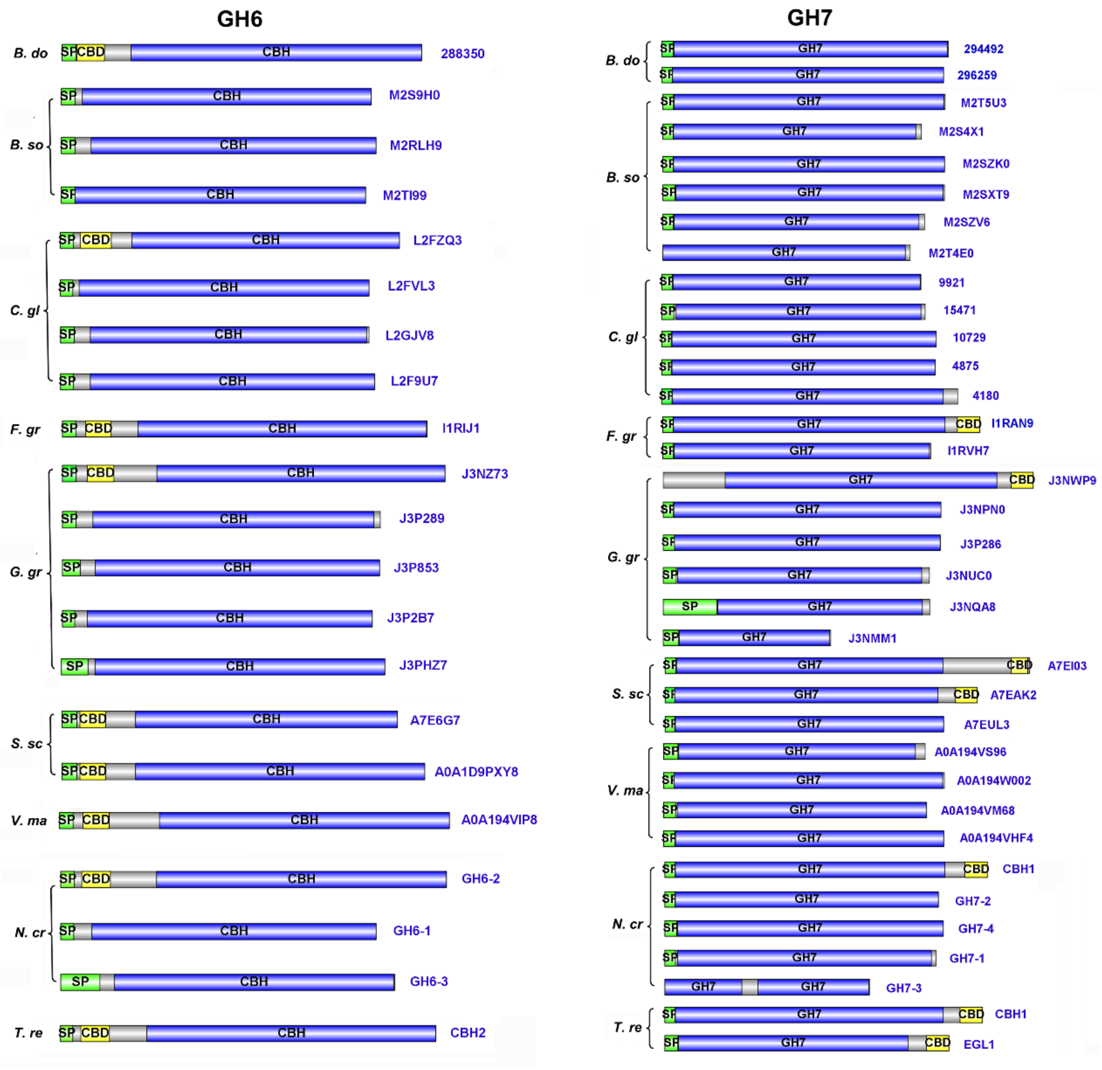

Figure S1. CBD organization in GH6 and GH7 cellulases.

1: S.Sc_A7EI03_GH7_CBD	100.00	48.39	40.62	50.00	43.75	28.12	40.62	40.62	37.50	50.00	43.75	37.50	37.50	34.38	43.75	40.62
2: S.Sc_A7EAK2_GH7_CBD	48.39	100.00	44.74	45.95	54.05	37.84	48.65	48.65	45.95	52.63	44.74	45.95	48.65	36.84	48.65	51.35
3: N.cr_CBH1_GH7_CBD	40.62	44.74	100.00	52.63	55.26	31.58	47.37	47.37	44.74	51.28	51.28	44.74	44.74	38.46	34.21	44.74
4: G.gr_J3NWP9_GH7_CBD	50.00	45.95	52.63	100.00	57.89	31.58	52.63	52.63	55.26	57.89	44.74	42.11	39.47	39.47	52.63	50.00
5: F.gr_I1RAN9_GH7_CBD	43.75	54.05	55.26	57.89	100.00	31.58	47.37	47.37	50.00	57.89	55.26	47.37	47.37	55.26	44.74	57.89
6: C.gl_L2FZQ3_GH6_CBD	28.12	37.84	31.58	31.58	31.58	100.00	47.37	47.37	36.84	36.84	36.84	57.89	60.53	44.74	50.00	50.00
7: S.Sc_A7E6G7_GH6_CBD	40.62	48.65	47.37	52.63	47.37	47.37	100.00	100.00	65.79	55.26	55.26	60.53	55.26	47.37	60.53	60.53
8: S.sc_A0A1D9PXY8_GH6_CBD	40.62	48.65	47.37	52.63	47.37	47.37	100.00	100.00	65.79	55.26	55.26	60.53	55.26	47.37	60.53	60.53
9: B.do_288350_GH6_CBD	37.50	45.95	44.74	55.26	50.00	36.84	65.79	65.79	100.00	52.63	47.37	52.63	44.74	47.37	57.89	55.26
10: T.re_CBH1_GH7_CBD	50.00	52.63	51.28	57.89	57.89	36.84	55.26	55.26	52.63	100.00	71.79	52.63	50.00	38.46	50.00	57.89
11: T.re_EGL1_GH7_CBD	43.75	44.74	51.28	44.74	55.26	36.84	55.26	55.26	47.37	71.79	100.00	55.26	55.26	51.28	55.26	63.16
12: N.cr_GH6-2_GH6_CBD	37.50	45.95	44.74	42.11	47.37	57.89	60.53	60.53	52.63	52.63	55.26	100.00	71.05	60.53	60.53	65.79
13: G.gr_J3NZ73_GH6_CBD	37.50	48.65	44.74	39.47	47.37	60.53	55.26	55.26	44.74	50.00	55.26	71.05	100.00	57.89	63.16	68.42
14: F.gr_I1RIJ1_GH6_CBD	34.38	36.84	38.46	39.47	55.26	44.74	47.37	47.37	47.37	38.46	51.28	60.53	57.89	100.00	52.63	68.42
15: V.ma_A0A194VIP8_GH6_CBD	43.75	48.65	34.21	52.63	44.74	50.00	60.53	60.53	57.89	50.00	55.26	60.53	63.16	52.63	100.00	68.42
16: T.re_CBH2_GH6_CBD	40.62	51.35	44.74	50.00	57.89	50.00	60.53	60.53	55.26	57.89	63.16	65.79	68.42	68.42	68.42	100.00

Figure S2. Identity in sequence alignment of CBDs from GH6 and GH7 cellulases.
